# Will COVID-19 be evidence-based medicine’s nemesis?

**DOI:** 10.1371/journal.pmed.1003266

**Published:** 2020-06-30

**Authors:** Trisha Greenhalgh

**Affiliations:** Nuffield Department of Primary Care Health Sciences, University of Oxford, Oxford, United Kingdom

Once defined in rhetorical but ultimately meaningless terms as “the conscientious, judicious and explicit use of current best evidence in making decisions about the care of individual patients” [1], evidence-based medicine rests on certain philosophical assumptions: a singular truth, ascertainable through empirical enquiry; a linear logic of causality in which interventions have particular effect sizes; rigour defined primarily in methodological terms (especially, a hierarchy of preferred study designs and tools for detecting bias); and a deconstructive approach to problem-solving (the evidence base is built by answering focused questions, typically framed as ‘PICO’—population-intervention-comparison-outcome) [2].

The trouble with pandemics is that these assumptions rarely hold. A pandemic-sized problem can be framed and contested in multiple ways. Some research questions around COVID-19, most notably relating to drugs and vaccines, are amenable to randomised controlled trials (and where such trials were possible, they were established with impressive speed and efficiency [3, 4]). But many knowledge gaps are broader and cannot be reduced to PICO-style questions. Were care home deaths avoidable [5]? Why did the global supply chain for personal protective equipment break down [6]? What role does health system resilience play in controlling the pandemic [7]? And so on.

Against these—and other—wider questions, the neat simplicity of a controlled, intervention-on versus intervention-off experiment designed to produce a definitive (i.e. statistically significant and widely generalisable) answer to a focused question rings hollow. In particular, upstream preventive public health interventions aimed at supporting widespread and sustained behaviour change across an entire population (as opposed to testing the impact of a short-term behaviour change in a select sample) rarely lend themselves to such a design [8, 9]. When implementing population-wide public health interventions—whether conventional measures such as diet or exercise, or COVID-19 related ones such as handwashing, social distancing and face coverings—we must not only persuade individuals to change their behavior but also adapt the environment to make such changes easier to make and sustain [10–12].

Population-wide public health efforts are typically iterative, locally-grown and path-dependent, and they have an established methodology for rapid evaluation and adaptation [9]. But evidence-based medicine has tended to classify such designs as “low methodological quality” [13]. Whilst this has been recognised as a problem in public health practice for some time [11], the inadequacy of the dominant paradigm has suddenly become mission-critical.

Whilst evidence-based medicine recognises that study designs must reflect the nature of question (randomized trials, for example, are preferred only for therapy questions [13]), even senior scientists sometimes over-apply its hierarchy of evidence. An interdisciplinary group of scholars from the UK’s prestigious Royal Society recently reviewed the use of face masks by the general public, drawing on evidence from laboratory science, mathematical modelling and policy studies [14]. The report was criticised by epidemiologists for being “non-systematic” and for recommending policy action in the absence of a quantitative estimate of effect size from robust randomized controlled trials [15].

Such criticisms appear to make two questionable assumptions: first, that the precise quantification of impact from this kind of intervention is both possible and desirable, and second, that unless we have randomized trial evidence, we should do nothing.

It is surely time to turn to a more fit-for-purpose scientific paradigm. Complex adaptive systems theory proposes that precise quantification of particular cause-effect relationships is both impossible (because such relationships are not constant and cannot be meaningfully isolated) and unnecessary (because what matters is what emerges in a particular real-world situation). This paradigm proposes that where multiple factors are interacting in dynamic and unpredictable ways, naturalistic methods and rapid-cycle evaluation are the preferred study design. The 20^th^-century logic of evidence-based medicine, in which scientists pursued the goals of certainty, predictability and linear causality, remains useful in some circumstances (for example, the drug and vaccine trials referred to above). But at a population and system level, we need to embrace 21^st^-century epistemology and methods to study how best to cope with uncertainty, unpredictability and non-linear causality [16].

In a complex system, the question driving scientific inquiry is not “what is the effect size and is it statistically significant once other variables have been controlled for?” but “does this intervention contribute, along with other factors, to a desirable outcome?”. Multiple interventions might each contribute to an overall beneficial effect through heterogeneous effects on disparate causal pathways, even though none would have a statistically significant impact on any predefined variable [11]. To illuminate such influences, we need to apply research designs that foreground dynamic interactions and emergence. These include in-depth, mixed-method case studies (primary research) and narrative reviews (secondary research) that tease out interconnections and highlight generative causality across the system [16, 17].

Table 1 lists some philosophical contrasts between the evidence-based medicine and complex-systems paradigms. Ogilvie et al have argued that rather than pitting these two paradigms against one another, they should be brought together [9]. As illustrated in (Fig 1), these authors depict randomized trials (what they call the “evidence-based practice pathway”) and natural experiments (the “practice-based evidence pathway”) in a complementary and recursive relationship rather than a hierarchical one. They propose that “…intervention studies [e.g. trials] should focus on reducing critical uncertainties, that non-randomised study designs should be embraced rather than tolerated and that a more nuanced approach to appraising the utility of diverse types of evidence is required.” (page 203) [9].

**Fig 1:**
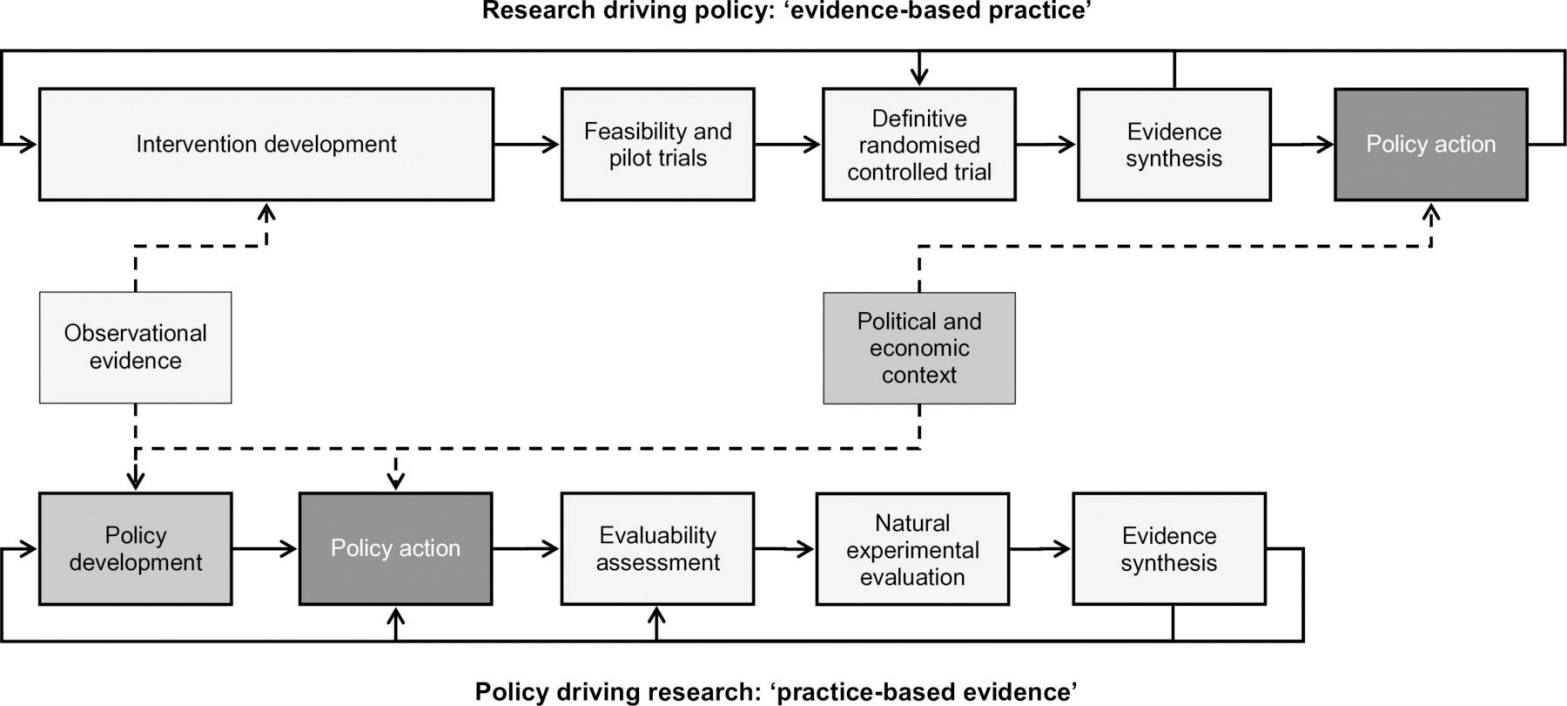
Ogilvie et al’s model of two complementary modes of evidence generation: evidence-based practice and practice-based evidence. Reproduced under CC-BY-4.0 licence from authors’ original [9].

**Table 1:** Evidence-based medicine versus complex systems research paradigms. Adapted under Creative Commons licence from Greenhalgh and Papoutsi [16].

	Evidence-based medicine paradigm	Complex systems paradigm
Perspective on scientific truth	Singular, independent of the observer, ascertainable through empirical inquiry	Multiple, influenced by mode of inquiry and perspective taken
Goal of research	Establishing the truth; finding more or less universal and generalisable solutions to well-defined problems	Exploring tensions; generating insights and wisdom; exposing multiple perspectives; viewing complex systems as moving targets
Assumed model of causality	Linear, cause-and-effect causality (perhaps incorporating mediators and moderators)	Emergent causality: multiple interacting influences account for a particular outcome but none can be said to have a fixed ‘effect size’
Typical format of research question	“What is the effect size of the intervention on the predefined outcome, and is it statistically significant?”	“What combination of influences has generated this phenomenon? What does the intervention of interest contribute? What happens to the system and its actors if we intervene in a particular way? What are the unintended consequences elsewhere in the system?”
Mode of representation	Attempt to represent science in one authoritative voice	Attempt to illustrate the plurality of voices inherent in the research and phenomena under study
Good research is characterised by	Methodological ‘rigour’, i.e. strict application of structured and standardised design, conventional approaches to generalisability and validity	Strong theory, flexible methods, pragmatic adaptation to emerging circumstances, contribution to generative learning and theoretical transferability
Purpose of theorising	Disjunctive: simplification and abstraction; breaking problems down into analysable parts	Conjunctive: drawing parts of the problem together to produce a rich, nuanced picture of what is going on and why
Approach to data	Research should continue until data collection is complete	Data will never be complete or perfect; decisions often need to be made despite incomplete or contested data
Analytic focus	Dualisms: A versus B; influence of X on Y	Dualities: inter-relationships and dynamic tensions between A, B, C and other emergent aspects

In the current fast-moving pandemic, where the cost of inaction is counted in the grim mortality figures announced daily, implementing new policy interventions in the absence of randomized trial evidence has become both a scientific and moral imperative. Whilst it is hard to predict anything in real time, history will one day tell us whether adherence to “evidence-based practice” helped or hindered the public health response to Covid-19—or whether an apparent slackening of standards to accommodate “practice-based evidence” was ultimately a more effective strategy.

## References

[1] Sackett DL, Rosenberg WM, Gray JM, Haynes RB, Richardson WS. Evidence based medicine: what it is and what it isn't. Bmj. 1996;312(7023):71–2. 10.1136/bmj.312.7023.71 8555924 PMC2349778

[2] Greenhalgh T. How to Read a Paper: The basics of evidence-based medicine and healthcare (6th edition). Oxford: John Wiley and Sons Ltd; 2019.

[3] Baden LR, Rubin EJ. Covid-19—the search for effective therapy. New Engl J Med. 2020;382:1851–2. 10.1056/NEJMe2005477 32187463 PMC7121446

[4] Lurie N, Saville M, Hatchett R, Halton J. Developing Covid-19 vaccines at pandemic speed. New Engl J Med. 2020;382:1969–73. 10.1056/NEJMp2005630 32227757

[5] Gordon AL, Goodman C, Achterberg W, Barker RO, Burns E, Hanratty B, et al COVID in Care Homes—Challenges and Dilemmas in Healthcare Delivery. Age and Ageing. 2020. 10.1093/ageing/afaa113 PMC7239229 32402088

[6] Armani AM, Hurt DE, Hwang D, McCarthy MC, Scholtz A. Low-tech solutions for the COVID-19 supply chain crisis. Nature Reviews Materials. 2020:1–4. 10.1038/s41578-020-0205-1 PMC7212509 32395258

[7] Legido-Quigley H, Asgari N, Teo YY, Leung GM, Oshitani H, Fukuda K, et al Are high-performing health systems resilient against the COVID-19 epidemic? The Lancet. 2020;395(10227):848–50. 10.1016/S0140-6736(20)30551-1 PMC7124523 32151326

[8] West R, Michie S, Rubin GJ, Amlôt R. Applying principles of behaviour change to reduce SARS-CoV-2 transmission. Nature Human Behaviour. 2020:1–9. 10.1038/s41562-020-0887-9 32377018

[9] Ogilvie D, Adams J, Bauman A, Gregg EW, Panter J, Siegel KR, et al Using natural experimental studies to guide public health action: turning the evidence-based medicine paradigm on its head. J Epidemiol Community Health. 2020;74(2):203–8. 10.1136/jech-2019-213085 31744848 PMC6993029

[10] Glass TA, McAtee MJ. Behavioral science at the crossroads in public health: extending horizons, envisioning the future. Social science & medicine. 2006;62(7):1650–71. 16198467 10.1016/j.socscimed.2005.08.044

[11] Rutter H, Savona N, Glonti K, Bibby J, Cummins S, Finegood DT, et al The need for a complex systems model of evidence for public health. The Lancet. 2017;390(10112):2602–4. 10.1016/S0140-6736(17)31267-9 28622953

[12] Jefferson T, Del Mar CB, Dooley L, Ferroni E, Al‐Ansary LA, Bawazeer GA, et al Physical interventions to interrupt or reduce the spread of respiratory viruses. Cochrane database of systematic reviews. 2011;(7). 10.1002/14651858.CD006207.pub4 PMC6993921 21735402

[13] Guyatt GH, Oxman AD, Vist GE, Kunz R, Falck-Ytter Y, Alonso-Coello P, et al GRADE: an emerging consensus on rating quality of evidence and strength of recommendations. Bmj. 2008;336(7650):924–6. 10.1136/bmj.39489.470347.AD 18436948 PMC2335261

[14] DELVE. Face Masks for the General Public. London: Royal Society DELVE (Data Evaluation and Learning for Viral Epidemics) initiative; 2020 Accessed 4th May 2020 at https://rs-delve.github.io/reports.html.

[15] Science Media Centre. Expert reaction to review of evidence on face masks and face coverings by the Royal Society DELVE Initiative. London: Science Media Centre; 2020 (4th May). Accessed 7th May 2020 at https://www.sciencemediacentre.org/expert-reaction-to-review-of-evidence-on-face-masks-and-face-coverings-by-the-royal-society-delve-initiative/.

[16] Greenhalgh T, Papoutsi C. Studying complexity in health services research: desperately seeking an overdue paradigm shift. BioMed Central; 2018 p. 95. 10.1186/s12916-018-1089-4 PMC6009054 29921272

[17] Greenhalgh T, Thorne S, Malterud K. Time to challenge the spurious hierarchy of systematic over narrative reviews? European journal of clinical investigation. 2018;48(6):e12931 10.1111/eci.12931 29578574 PMC6001568

